# General anaesthesia and deep sedation for monopolar pulsed field ablation using a lattice-tip catheter combined with a novel three-dimensional mapping system

**DOI:** 10.1093/europace/euae270

**Published:** 2024-11-22

**Authors:** Andreas Rillig, Jun Hirokami, Fabian Moser, Stefano Bordignon, Laura Rottner, Tohoku Shota, Ilaria My, Andrea Urbani, Marc Lemoine, Joseph Kheir, Niklas Schenker, Lukas Urbanek, Katarina Govorov, David Schaack, Julius Obergassel, Jan Riess, Djemail Ismaili, Paulus Kirchhof, Feifan Ouyang, Boris Schmidt, Bruno Reissmann, Kyoung-Ryul Julian Chun, Andreas Metzner

**Affiliations:** Department of Cardiology, University Heart and Vascular Center Hamburg-Eppendorf, Martinistr. 51, 20251 Hamburg, Germany; Cardioangiologisches Centrum Bethanien (CCB), Frankfurt Academy For Arrhythmias (FAFA), Frankfurt am Main, Germany; Abteilung für Kardiologie, Medizinische Klinik III, Agaplesion Markus Krankenhaus, Frankfurt am Main, Germany; Department of Cardiology, University Heart and Vascular Center Hamburg-Eppendorf, Martinistr. 51, 20251 Hamburg, Germany; Cardioangiologisches Centrum Bethanien (CCB), Frankfurt Academy For Arrhythmias (FAFA), Frankfurt am Main, Germany; Abteilung für Kardiologie, Medizinische Klinik III, Agaplesion Markus Krankenhaus, Frankfurt am Main, Germany; Department of Cardiology, University Heart and Vascular Center Hamburg-Eppendorf, Martinistr. 51, 20251 Hamburg, Germany; Cardioangiologisches Centrum Bethanien (CCB), Frankfurt Academy For Arrhythmias (FAFA), Frankfurt am Main, Germany; Abteilung für Kardiologie, Medizinische Klinik III, Agaplesion Markus Krankenhaus, Frankfurt am Main, Germany; Department of Cardiology, University Heart and Vascular Center Hamburg-Eppendorf, Martinistr. 51, 20251 Hamburg, Germany; Cardioangiologisches Centrum Bethanien (CCB), Frankfurt Academy For Arrhythmias (FAFA), Frankfurt am Main, Germany; Abteilung für Kardiologie, Medizinische Klinik III, Agaplesion Markus Krankenhaus, Frankfurt am Main, Germany; Department of Cardiology, University Heart and Vascular Center Hamburg-Eppendorf, Martinistr. 51, 20251 Hamburg, Germany; Cardioangiologisches Centrum Bethanien (CCB), Frankfurt Academy For Arrhythmias (FAFA), Frankfurt am Main, Germany; Abteilung für Kardiologie, Medizinische Klinik III, Agaplesion Markus Krankenhaus, Frankfurt am Main, Germany; Department of Cardiology, University Heart and Vascular Center Hamburg-Eppendorf, Martinistr. 51, 20251 Hamburg, Germany; Cardioangiologisches Centrum Bethanien (CCB), Frankfurt Academy For Arrhythmias (FAFA), Frankfurt am Main, Germany; Abteilung für Kardiologie, Medizinische Klinik III, Agaplesion Markus Krankenhaus, Frankfurt am Main, Germany; Department of Cardiology, University Heart and Vascular Center Hamburg-Eppendorf, Martinistr. 51, 20251 Hamburg, Germany; Cardioangiologisches Centrum Bethanien (CCB), Frankfurt Academy For Arrhythmias (FAFA), Frankfurt am Main, Germany; Abteilung für Kardiologie, Medizinische Klinik III, Agaplesion Markus Krankenhaus, Frankfurt am Main, Germany; Department of Cardiology, University Heart and Vascular Center Hamburg-Eppendorf, Martinistr. 51, 20251 Hamburg, Germany; Department of Cardiology, University Heart and Vascular Center Hamburg-Eppendorf, Martinistr. 51, 20251 Hamburg, Germany; Department of Cardiology, University Heart and Vascular Center Hamburg-Eppendorf, Martinistr. 51, 20251 Hamburg, Germany; Department of Cardiology, University Heart and Vascular Center Hamburg-Eppendorf, Martinistr. 51, 20251 Hamburg, Germany; Department of Cardiology, University Heart and Vascular Center Hamburg-Eppendorf, Martinistr. 51, 20251 Hamburg, Germany; Cardioangiologisches Centrum Bethanien (CCB), Frankfurt Academy For Arrhythmias (FAFA), Frankfurt am Main, Germany; Abteilung für Kardiologie, Medizinische Klinik III, Agaplesion Markus Krankenhaus, Frankfurt am Main, Germany; Department of Cardiology, University Heart and Vascular Center Hamburg-Eppendorf, Martinistr. 51, 20251 Hamburg, Germany; Cardioangiologisches Centrum Bethanien (CCB), Frankfurt Academy For Arrhythmias (FAFA), Frankfurt am Main, Germany; Abteilung für Kardiologie, Medizinische Klinik III, Agaplesion Markus Krankenhaus, Frankfurt am Main, Germany; Department of Cardiology, University Heart and Vascular Center Hamburg-Eppendorf, Martinistr. 51, 20251 Hamburg, Germany

**Keywords:** Atrial fibrillation ablation, Pulsed field ablation, Monopolar, Lattice tip, 3D mapping, Anaesthesia, Deep sedation, General anaesthesia

## Abstract

**Aims:**

A novel three-dimensional mapping platform combined with a lattice-tip catheter that can toggle between monopolar pulsed field ablation (PFA) and radiofrequency energy delivery was recently launched. So far, the system was predominantly applied in general anaesthesia (GA), not in deep sedation.

**Methods and results:**

Patients with symptomatic paroxysmal or persistent atrial fibrillation (AF) were enrolled, and pulmonary vein isolation (PVI) and ablation of additional linear lesion sets were performed either in GA or in deep sedation. Pulsed field ablation was applied exclusively to perform ipsilateral PVI. A total of 63 patients (35% female, 75% persistent AF, mean age 64 ± 9 years) were included in the analysis with 23 patients treated in GA and 40 patients in deep sedation. Acute efficacy was comparable in both groups with a PVI rate of 100%. Additional 74 lesion sets were performed in the total cohort. Mean procedure and lab occupancy time in the GA and deep sedation group was 96 ± 24 min vs. 100 ± 23 min (*P* = 0.52) and 165 ± 40 min vs. 131 ± 35 min (*P* = 0.0008). Mean dose area product was 489 (216;1093) vs. 452 (272;882) cGycm^2^ in the GA and the deep sedation group (*P* = 0.82). There was one conversion from deep sedation to GA. There were no map shifts observed in any group. Pericardial tamponade occurred in one patient of the deep sedation group.

**Conclusion:**

The use of a novel ablation platform in conjunction with a lattice-tip catheter in deep sedation is feasible, effective, and associated with significantly shorter lab occupancy time when compared with GA.

## Graphical Abstract

**Graphical Abstract:**
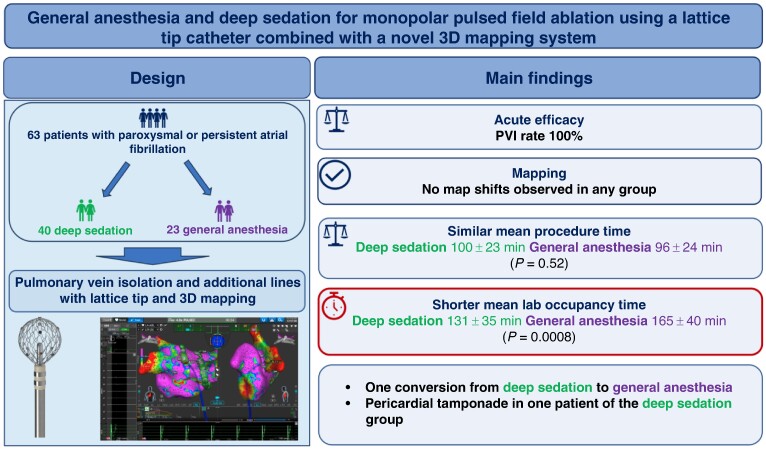

**What’s new?**

- In atrial fibrillation patients ablated with the novel lattice-tip catheter in conjunct with a three-dimensional mapping system using either pulsed field ablation or radiofrequency, no significant differences in procedure or fluoroscopy times were observed between deep sedation and general anaesthesia (GA).
- Whereas acute procedural efficacy was high and comparable between both groups, lab occupancy time was shorter when procedures were performed in deep sedation only.
- There were no map shifts in both treatment groups, and conversion from deep sedation to GA was necessary in only one single patient.

## Introduction

**Figure 1:**
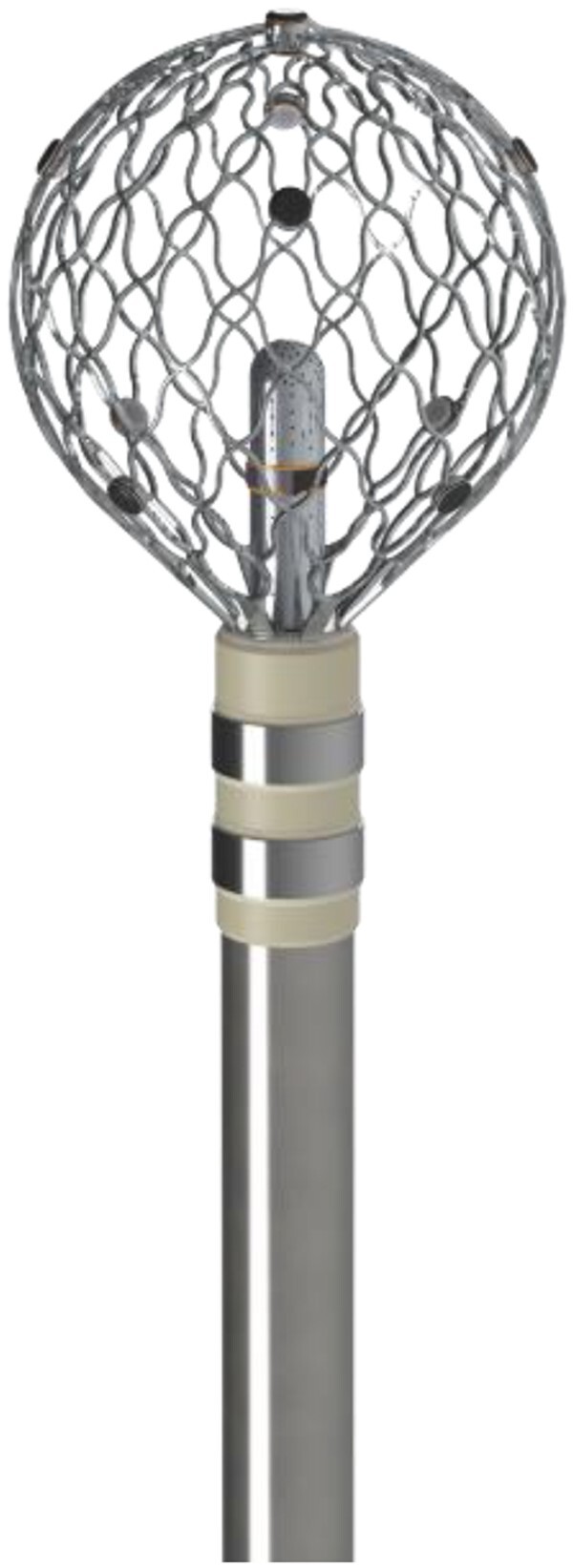
The novel lattice-tip catheter with a diameter of 9 mm with nine mini-electrodes at its surface and a central electrode. The catheter is bidirectionally steerable and can toggle between pulsed field and RF ablation.

Pulsed field ablation (PFA) is a novel non-thermal ablation modality introduced into the field of cardiac arrhythmias.^1^ Expectations regarding acute and long-term efficacy are high, same as for safety due to the selectiveness of PFA for myocardial cells.^1–4^ Recently, a novel mapping and ablation platform (Affera™, Medtronic) combined with a new mapping and ablation catheter (Sphere-9™, Medtronic) was introduced.^5–8^ The tip of this unique irrigated mapping and ablation catheter is lattice shaped with nine mini-electrodes on its surface and one central electrode (*Figure 1*). This catheter can apply PFA as well as radiofrequency (RF) according to anatomical locations and operator’s choice and yields high flexibility for individual ablation strategies.^5,6,9^ So far, Affera-based ablation was only performed in general anaesthesia (GA), and there is no experience for procedures in deep sedation.^5,6,9^ However, in Europe and the USA, conscious or deep sedation is mainly used for electrophysiology procedures.^10,11^ Catheter ablation in deep sedation using fentanyl, midazolam, and a ketamine adjunct or midazolam, dexmedetomidine, and remifentanil was recently described for ablation with the Farapulse PFA catheter (Boston Scientific) or a variable loop circular PFA catheter (Biosense Webster).^12,13^

The present study compared the safety and efficacy of Affera procedures with PFA and/or RF applications in atrial fibrillation (AF) patients ablated in deep sedation or in GA.

## Methods

### Inclusion and exclusion criteria

Patients with symptomatic paroxysmal or persistent AF and no previous left atrial (LA) ablation attempt were included. Exclusion criteria were a LA diameter > 55 mm, evidence of a LA thrombus, and contraindication to anticoagulation.

This retrospective, multicentre study conducted at two high-volume AF ablation centres in Germany was approved by each centre’s ethics committee/institutional review board and conducted in accordance with the Declaration of Helsinki. Informed consent was obtained from all participants. Data of this study are available upon reasonable request.

### Pre-procedural management

Transoesophageal echocardiography was performed in all patients prior to ablation to rule out LA thrombus. In patients on vitamin K antagonists, ablation was performed under therapeutic INR values ranging between 2 and 3. Novel oral anticoagulants were stopped at the day of the procedure and were resumed 6 h post-ablation.

### The novel mapping and ablation platform

The mapping and ablation system (Affera™) consists of the HexaGEN™ RF generator, the HexaPULSE™ pulsed field (PF) generator module, and the HexaFLOW™ irrigation pump, along with additional accessories that are designed for use with compatible ablation catheters (Sphere-9™) to treat cardiac arrhythmias.

With the temperature-controlled generator, HexaGen™, the system delivers RF energy to create wide-area focal RF lesions. Pulsed field lesions are generated by utilizing the high-voltage PFA energy generator, HexaPulse™. The HexaMap™ catheter interface unit provides magnetic navigation and map acquisition with the lattice-tip catheter (Sphere-9™). It displays and records intra-cardiac electrograms (EGMs) with an internal pacing functionality. The PRISM-1 software provides three-dimensional (3D) mapping acquisition.

The ablation catheter is 8 Fr in diameter and bidirectional with a built-in controller for direct mapping system interaction, allowing mapping and ablation with dual-energy capabilities, i.e. RF and PFA. Its nine mini-electrodes and one central electrode provide high-density mapping capabilities, utilizing high-fidelity, close-unipolar EGMs. The 9 mm conformable lattice tip is designed to improve tip-to-tissue contact and catheter stability.

The system allows temperature-controlled RF delivery through the whole lattice, which serves as a continuous conductive ablation electrode with an effective surface area 10× larger than standard irrigated ablation catheters. In terms of PFA energy delivery, the lattice-tip catheter delivers a proprietary unipolar, biphasic waveform to cause cell death by irreversible electroporation. As with RF, the entire ablation electrode delivers PFA energy consisting of a train of 12 ms trains with 1500 pulses each.

### General anaesthesia and deep sedation protocol

General anaesthesia was conducted by experienced cardiac anaesthesiologists. General anaesthesia was induced by continuous remifentanil infusion, a bolus of propofol (1% solution) and succinylcholine or rocuronium for intubation. Patients’ lungs were mechanically ventilated *via* an endotracheal tube, and GA was maintained with continuous propofol and remifentanil infusion. All patients were given balanced electrolyte solution at the discretion of the treating anaesthesiologists, and continuous infusion of noradrenaline was administered to prevent hypotension. On demand of the electrophysiologists, deep muscle relaxation was induced by a bolus of rocuronium. At the end of the procedure, residual muscle relaxation was antagonized with neostigmine or sugammadex. After extubation, the patients were surveilled in the peri-operative anaesthesia care unit.

Deep sedation was managed by trained staff of the electrophysiology laboratories and initiated by a bolus of fentanyl (25 µg) and propofol (1% solution) followed by a continuous propofol infusion. Propofol was usually started with a bolus between 3 and 5 mg and continued with an infusion rate of 5 mg/KG/h. No anaesthetist was in the room during deep sedation. Before PFA or RF application, an additional bolus of fentanyl (25 µg) was applied as well as propofol boluses if necessary. Propofol flow rate was adapted according to patient’s body weight and sedation level: if patient movement during energy application or blood pressure rise was observed, propofol flow rate was carefully increased and/or an additional propofol bolus was applied.

### Ablation procedure

After ultrasound-guided puncture of the right femoral vein, a diagnostic catheter was positioned inside the coronary sinus. Following single or double transseptal puncture guided by fluoroscopy and using a modified Brockenbrough technique, a single transseptal sheath (SL1, Abbott) was advanced into the LA. After transseptal puncture, intravenous heparin was administered to obtain an activated clotting time > 300 s. Selective pulmonary vein (PV) angiography was performed to identify the PV ostia. As soon as the activated clotting time level was ≥300 s, the ablation catheter was introduced and a 3D bipolar voltage map of the LA and the PVs was created. In order to precisely define the ridge between the lateral PVs and the LA appendage, a second map of the lateral PVs only was acquired and merged with the LA map. The ostia of the ipsilateral PVs were tagged according to PV angiographies and local electrical information.

### Pulmonary vein isolation

**Figure 2:**
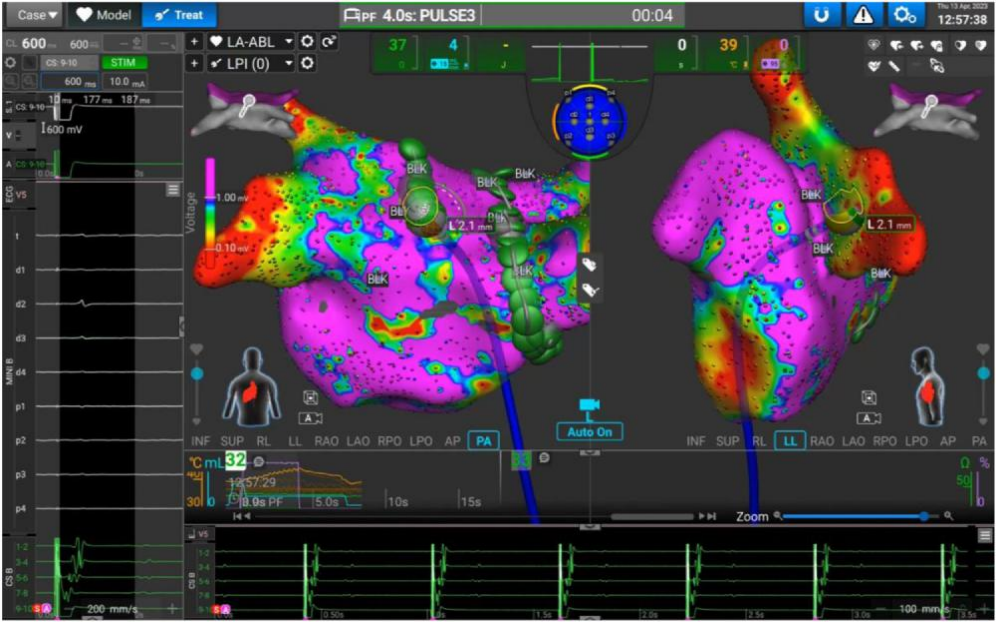
Three-dimensional bipolar map of the LA and the right- and left-sided PVs in a posterior–anterior and a left lateral projection. The green dots mark PFA-based lesions around the ipsilateral PVs as applied by the lattice-tip mapping and ablation catheter.

In the current series, PFA only was applied around the PV ostia to isolate the PVs. The catheter was guided point by point around the ipsilateral PVs, and PFA was applied for a duration of 4 s each. The inter-lesion distance, automatically measured by the system, was kept at a maximum of 5 mm (*Figure 2*). After completion of both ipsilateral circles, another voltage map of the LA and the PVs was generated and PV isolation (PVI) was evaluated according to bipolar information and the absence of PV potentials on the lattice-tip catheter along the ablation line and by a spiral mapping catheter, when used. In case of catheter instability and requirement for more mechanical support, a steerable sheath (Vizigo™, Biosense Webster) was used at the discretion of the operator.

### Ablation of additional linear lesion sets beyond the pulmonary veins

In patients with extensive LA low voltage areas or with additional atrial tachycardia, linear lesions were applied at operator’s discretion. If linear lesion sets at the posterior LA wall (roof line or posterior wall isolation) were performed, only PFA was used. Along the anterior wall and the mitral isthmus area, RF or PFA was applied at operator’s discretion. In patients with documentation of typical cavo-tricuspid isthmus (CTI)-dependent atrial flutter, the CTI was targeted using RF.

### Post-procedural care

Following ablation, all patients underwent transthoracic echocardiography to rule out pericardial effusion. All patients were treated with proton-pump inhibitors for 6 weeks. Novel oral anticoagulants were resumed 6 h post-ablation. Anticoagulation was continued for at least 3 months and, thereafter, based on the individual CHA_2_DS_2_-VASc score.

### Statistical analysis

Continuous data are described as means and standard deviations in normally distributed data, or as medians, 25th and 75th percentiles otherwise. Categorical data are described with absolute and relative frequencies. A two-sided *P*-value <0.05 was considered statistically significant.

## Results

### Baseline characteristics

A total of 63 consecutive patients (65% male, 64 ± 9 years, 75% persistent AF) were included into our analysis. All baseline characteristics are depicted in *Table 1*. While a total of 16 (25%) patients were treated in GA with the application of muscle relaxants, another 7 (11%) patients were treated in GA without muscle relaxants, and finally, 40 (63%) patients were treated in deep sedation only.

**Table 1:** Baseline characteristics

	Total (*n* = 63)	General anaesthesia (*n* = 23)	Deep sedation (*n* = 40)	*P*-value
Age (years)	64 ± 9	68 ± 8	65 ± 10	0.223
Male, *n* (%)	41 (65)	14 (61)	27 (68)	0.602
Persistent atrial fibrillation, *n* (%)	47 (75)	14 (61)	33 (83)	0.061
Body mass index	28 ± 5	28 ± 6	28 ± 5	0.84
Left ventricular ejection fraction (%)	56 ± 9	55 ± 8	57 ± 9	0.526
Left atrial diameter (mm)	42 ± 5	42 ± 5	43 ± 4	0.875
CHA_2_DS_2_VASc score	2 (1;4)	3 (2;4)	2 (1;4)	0.664
Hypertension, *n* (%)	40 (63)	15 (65)	25 (63)	0.833
Coronary artery disease, *n* (%)	12 (19)	4 (17)	8 (20)	0.804
Diabetes mellitus, *n* (%)	7 (11)	3 (13)	4 (10)	0.717
History of transitory ischaemic attack/stroke, *n* (%)	7 (11)	5 (22)	2 (5)	**0**.**043**

Bold number indicate statistically significant value.

### Acute ablation results for general anaesthesia and deep sedation

In all patients, high-density voltage maps of the LA were generated with a mean of 4051 ± 1100 EGMs (3997 ± 1019 and 4079 ± 1152 EGMs in the GA and the deep sedation group) per map and a mean LA volume of 163 ± 41 mL (156 ± 40 mL and 166 ± 42 mL for GA and deep sedation).

All PVs were successfully isolated with a first-pass isolation rate of 99%. Mean number of applications and total application time were 43 ± 10 and 174 ± 40 s for the septal PVs and 42 ± 9 and 166 ± 37 s for the lateral PVs in the GA group and 46 ± 13 and 184 ± 54 s for the septal PVs and 46 ± 11 and 183 ± 45 s for the lateral PVs in the deep sedation group (*Table 2*).

**Table 2:** Ablation characteristics of PVI

	All patients (*n* = 63)	General anaesthesia (*n* = 23)	Deep sedation (*n* = 40)	*P*-value
Septal pulmonary veins (*n*)	62			
PFA-appl., *n*	45 ± 12	43 ± 10	46 ± 13	0.399
Application time (s)	181 ± 49	174 ± 40	184 ± 54	0.410
First-pass isolation, *n* (%)	61 (98)	23 (100)	39 (98)	0.283
Lateral pulmonary veins (*n*)	63			
PFA-appl., *n*	44 ± 11	42 ± 9	46 ± 11	0.134
Application time (s)	177 ± 43	166 ± 37	183 ± 45	0.147
First-pass isolation, *n* (%)	61 (100)	23 (100)	47 (100)	1
Total procedure time (min)	98 ± 23	96 ± 24	100 ± 23	0.517
Lab occupancy time (min)	143 ± 40	165 ± 40	131 ± 35	**0**.**0008**
Fluoroscopy time (min)	8.7 ± 3.4	8.1 ± 2.2	9.1 ± 3.9	0.262
Dose area product (cGcm^2^)	454 (249;998)	489 (216;1093)	452 (272;882)	0.824

Bold number indicate statistically significant value.

A total of 19 additional linear lesion sets (11 roof lines, 1 posterior wall lesion, 4 anterior lines, 1 mitral isthmus line, and 2 CTI lines) were applied in the 23 patients of the GA group (mean of 0.8 additional linear lesions per patient) and 55 additional linear lesion sets (26 roof lines, 0 posterior box lesion, 18 anterior lines, 3 mitral isthmus lines, and 7 CTI lines) in the 40 patients of the deep sedation group (mean of 1.3 additional linear lesions per patient).

All linear lesion sets were finally bidirectionally blocked, and the posterior box lesion electrically isolated.

Mean procedure time was 96 ± 24 min and 100 ± 23 min (*P* = 0.517) and mean lab occupancy time 165 ± 40 min and 131 ± 35 min (*P* = 0.0008) for the GA and the deep sedation group. Mean fluoroscopy time and dose area product was 8.1 ± 2.2 vs. 9.1 ± 3.9 min (*P* = 0.262) and 489 (216;1093) vs. 452 (272;882) cGycm^2^ (*P* = 0.824) for the GA and the deep sedation group, respectively.

Of note, in none of the procedures performed in deep sedation, any map shifts or significant patient movements occurred potentially compromising the procedural outcome. In only 1/40 (3%) patients treated in deep sedation, conversion to larynx mask and ventilation was necessary. The patient was obese (BMI > 40 kg/m^2^) and the tongue repeatedly slipped back and blocked the airways.

### Complications

No device-specific complications occurred in the total cohort. In 1/40 (3%) patients of the deep sedation group, cardiac tamponade occurred immediately after transseptal puncture before introducing the Sphere-9 catheter into the LA. The tamponade was treated by pericardial puncture and the patient recovered without any sequelae. No stroke or transitory ischaemic attack, no air embolism, no clinically apparent coronary spasm, no phrenic nerve paralysis, and no access site complication occurred in any of the patients (*Table 3*).

**Table 3:** Complications

	All patients (*n* = 63)	General anaesthesia (*n* = 23)	Deep sedation (*n* = 40)
Transitory ischaemic attack/stroke, *n* (%)	0	0	0
Pericardial effusion, *n* (%)	0	0	0
Pericardial tamponade, *n* (%)	1 (2)	0	1 (3)
Air embolism, *n* (%)	0	0	0
Coronary spasm, *n* (%)	0	0	0
Access site complication, *n* (%)	0	0	0
Phrenic nerve palsy, *n* (%)	0	0	0

## Discussion

The current study shows important insights from the first commercially performed AF ablation procedures applying a novel mapping and ablation platform combined with a lattice-tip catheter in deep sedation as compared with GA. The study found that:

1. No significant differences in procedure or fluoroscopy times in patients ablated in deep sedation when compared with GA were observed. Importantly, the lab occupancy time was shorter when procedures were performed in deep sedation only.
2. There were no map shifts in both treatment groups, and conversion from deep sedation to GA was necessary in only one single patient.
3. Acute procedural efficacy was comparable between both groups.

Catheter ablation is an effective treatment for AF. However, RF and cryoenergy as the most established energy forms are limited by reasonable drawbacks mainly based on non-selective thermal tissue effects or catheter design.^14^ Therefore, technologies adopting the benefits and overcoming the limitations of current ablation technologies are needed. The novel Affera 3D mapping and ablation platform integrates a unique catheter with a conformable lattice tip. This catheter combines high-resolution mapping properties and stable and broad tissue contact, improved ablation characteristics by optimized energy transfer and broad ablation lesions, and allows for both application of PFA and RF.^6,9^ While myocardial cells are highly sensitive to PFA, other cardiac cell types and tissues such as the vasculature, nerve cells, and extracardiac structures such as the oesophagus or the phrenic nerve are less PFA sensitive and thus protected against this type of energy application. At the same time, PFA is acutely and clinically highly effective.^14^

General anaesthesia might have advantages over deep sedation during AF ablation.^15^ Due to less thoracic movement during controlled ventilation, improved catheter stability and potentially less 3D map shifts might result. As a consequence, shorter ablation times and less energy applications were demonstrated.^15^ However, GA makes ablation procedures and settings more complex and time-consuming with longer lab occupancy times and potentially increases procedure costs. Although some benefits of AF ablation in GA have been described, the vast majority of AF ablation procedures are performed in deep sedation and conscious sedation in Europe or the USA. The advent of PFA has led to discussions about what sedation strategy is most useful for AF ablation with this technology and whether GA might be mandatory.^10^ There have been several reports on the feasibility of different PFA ablation tools during deep sedation.^12,13^ Atrial fibrillation ablation in deep sedation using PFA was feasible using the Centauri (CardioFocus) system with its monopolar energy delivery^16^ as well as with the bipolar Farapulse catheter (Boston Scientific) and the bipolar variable loop catheter (Biosense Webster).^12,13^ However, the novel Affera system with its lattice-tip catheter has energy settings different to the beforementioned systems, and due to the lattice-tip catheter design, ablation in conjunct with a 3D mapping is necessary. This raised concerns that catheter ablation of AF without GA using this monopolar system might result in map shifts leading to imperfect lesion creation, prolonged procedure, and fluoroscopy times or safety compromise. This two-centre experience shows that circumferential PVI using PFA as well as application of different lesion sets within the left and right atrium was feasible with deep sedation using propofol and fentanyl. No map shifts occurred and the acute PV isolation rate and first-pass isolation rate were comparable in both groups. At the same time, lab occupancy times were shorter when procedures were performed in deep sedation only. This observation is of major importance since lab capacities in most EP centres are limited and waiting lists of patients seeking for ablation are long. Candidates in whom GA instead of deep sedation should be considered are obese patients with BMIs > 35 kg/m^2^. No safety issues have been observed.

Therefore, deep sedation can be considered a viable option for left and right atrial ablation using the Affera system and the lattice-tip Sphere-9 catheter.

### Limitations

In the current retrospective observational series, only a limited number of patients were treated. No systematic clinical follow-up is available yet but was not the scope of the analysis and will be provided in future studies. Although patient groups were similar, selection bias cannot be definitely ruled out. Patient satisfaction was not captured in this patient population and requires further evaluation.

## Conclusion

Pulmonary vein isolation with the novel Affera mapping platform and the lattice-tip ablation catheter can be performed in deep sedation with high primary efficacy. Lab occupancy times can be reduced, when deep sedation is used instead of GA.

## Data Availability

Data are available on reasonable request.
